# LncRNA Expression Profiles in C6 Ceramide Treatment Reveal lnc_025370 as a Promoter in Canine Mammary Carcinoma CHMp Cells Progression

**DOI:** 10.3390/cimb46120849

**Published:** 2024-12-16

**Authors:** Hongxiu Diao, Fangying Zhao, Meijin Wu, Yan Zhang, Qianting Tao, Shichao Chen, Degui Lin

**Affiliations:** 1 Joint Laboratory of Animal Pathogen Prevention and Control of Fujian-Nepal, College of Animal Sciences, Fujian Agriculture and Forestry University, Fuzhou 350002, China; diaohongxiu@yeah.net (H.D.); 13338528861@163.com (M.W.); mszyzhangyan@foxmail.com (Y.Z.); 13394040904@163.com (S.C.); 2 Fujian Province Joint Laboratory of Animal Pathogen Prevention and Control of the “Belt and Road”, College of Animal Sciences, Fujian Agriculture and Forestry University, Fuzhou 350002, China; 3 Department of Clinical Veterinary Medicine, College of Veterinary Medicine, China Agricultural University, Beijing 100193, China; zhaofy97@126.com

**Keywords:** canine mammary carcinomas, C6 ceramide, Lnc_025370, NRG1, expression profiles

## Abstract

Canine mammary carcinomas (CMCs) represent the most prevalent form of cancer in female dogs, characterized by a high incidence and mortality rate. C6 ceramide is recognized for its multifaceted anti-cancer properties, yet its specific influence on CMCs remains to be elucidated. Long noncoding RNAs (lncRNAs), now recognized as functional “dark matter” in precision oncology, are particularly intriguing, with 44% of canine lncRNAs exhibiting tissue-specific expression. In this study, we performed a thorough analysis of lncRNA expression profiles to uncover the mechanisms behind C6 ceramide’s anti-cancer activity in CHMp cells. Our findings reveal that C6 ceramide notably inhibits the proliferation of CHMp cells. RNA sequencing identified 4522 lncRNAs with expression changes following C6 ceramide treatment, of which 2936 were upregulated and 1586 were downregulated. Further investigation into Lnc_025370 showed that it is predominantly nuclear-localized and is significantly downregulated by C6 ceramide treatment. Functional studies discovered that overexpression of Lnc_025370 enhances the growth and metastatic capabilities of CHMp cells, which is associated with an increase in NRG1, and concurrently diminishes the anti-cancer effectiveness of C6 ceramide in vitro. Mouse xenograft models also showed that Lnc_025370 overexpression promotes tumor growth and Ki67 expression. Together, our results suggest that Lnc_025370 acts as a pivotal target mediator of C6 ceramide’s anti-cancer effects, facilitating the malignant progression of CHMp cells.

## 1. Introduction

Canine mammary carcinomas (CMCs) are the most common tumors in female dogs, characterized by high rates of metastasis and mortality. Additionally, the incidence of CMCs tends to be associated with age, breed, and spayed status. The incidence of CMCs in unneutered female dogs accounts for 75.6−80.3% of observed tumors [1,2], and approximately 50% of these cases are malignant [3,4]. The age group between 9–12 years has the highest incidence rates, followed by 5–8-year-old female dogs [1]. Currently, multiple approaches, including surgical resection, chemotherapy, radiation therapy, or their combinations, are utilized to cure or control CMCs. However, high rates of recurrence and/or metastases remain a significant clinical problem for some patients, which can greatly reduce a dog’s quality of life and pose a threat to their overall health and well-being [5,6]. Therefore, the main challenge in improving CMC management is finding new therapeutic strategies to effectively treat the disease or diagnosis early.

C6 ceramide, a type of sphingolipid metabolite, has been found to inhibit breast cancer proliferation and metastasis by inducing apoptosis in humans, either alone or in combination with chemotherapy drugs [7,8,9,10]. Currently, various ceramide-based cancer therapies are under investigation in preclinical and clinical trials (phase I and II) to treat human breast cancer [11]. CMC is highly comparable to human breast cancer in terms of spontaneous development, environmental factors, epidemiology, pathological features, subtype classification, and molecular characteristics [3,12,13]. Therefore, it can be an ideal animal model for researching human breast cancer. Given the similarities between CMCs and human breast cancers, C6 ceramide may also prove beneficial in treating CMCs.

Long noncoding RNAs (lncRNAs), once considered nonfunctional “garbage” have now been redefined as functional “dark matter”, particularly in precision oncology. Their oncogenic role and higher expression in various cancer types compared to adjacent tissues have sparked significant clinical interest in using lncRNAs as diagnostic and prognostic biomarkers [14,15,16]. Liu et al. found that lncRNA ROPM was upregulated in breast cancer patients and targeted PLA2G16 to govern breast cancer stem cell properties, resulting in a positive correlation with malignant grade/stage and poor prognosis [17]. Similarly, NEAT1 alters metabolism to enhance breast cancer growth and metastasis [18]. Conversely, LncRNA-BC069792 suppresses breast cancer progression by targeting KCNQ4 to inactivate JAK2 and p-AKT [19]. Unlike human breast cancer, research on the role of lncRNAs in CMCs is rare. Currently, thousands of lncRNAs related to CMCs have been discovered, but research on their functional mechanisms remains scarce [20,21]. It is worth noting that 44% of canine lncRNAs are exhibited in a tissue-specific manner [20], and this enhances their potential to be biomarkers for CMCs [22].

In this study, we used RNA-sequencing to identify differentially expressed lncRNAs following C6 ceramide treatment in canine mammary carcinoma CHMp cells. Subsequently, we conducted in vitro and in vivo experiments to confirm the regulatory role and to explore the potential mechanisms of a specific lncRNA in canine mammary carcinoma CHMp cells. The ultimate goal of this research is to identify new therapeutic targets aimed at treating canine mammary carcinoma.

## 2. Materials and Methods

### 2.1. Cell Lines and Cell Culture

The CHMp cell line, generously donated by the Graduate School of Agricultural and Life Sciences at the University of Tokyo, originated from an in situ mammary carcinoma in a 12-year-old female dog of mixed breed. Clinically, the tumor was staged as T4N1+M1, and histological analysis confirmed it as an inflammatory adenocarcinoma. Cells were cultured in DMEM supplemented with 10% fetal bovine serum (FBS), penicillin (100 U/mL), and streptomycin (100 μg/mL), and incubated in a humidified atmosphere with 5% CO_2_ at 37 °C.

### 2.2. Cell Viability Assay

Proliferation of CHMp cells was quantified using a Cell Counting Kit-8 (CCK-8) (Beyotime Institute of Biotechnology, Shanghai, China). CHMp cells were plated in 96-well plates at 1 × 10^4^ cells/well and incubated overnight to allow attachment. Cells were treated with different concentrations of C6 ceramide (10 μM, 20 μM, 30 μM, and 40 μM) (USA and Cayman Chemical, MI, USA) for 48 h. CCK-8 (10 μL) was added to each well, and following 1 h of incubation, the optical density was read at 450 nm with a microplate reader (Agilent Technologies, Santa Clara, CA, USA). Each group had five biological replicates.

### 2.3. Colony Formation Assay

Cells were plated in 6-well plates at 200 cells/well and incubated overnight to allow attachment. Then, the cells were treated with 6 and 10 μM C6 ceramide for 48 h, respectively. After treatment, the plates were washed and cultured with 10% FBS DMEM for 8–10 days. The attached cells were stained with 0.1% (*w*/*v*) crystal violet and the wells were photographed. Each group had three biological replicates.

### 2.4. Sample Process and Transcriptome Sequencing

Cells were plated in 6-well plates at 8 × 10^4^ cells/well and incubated overnight to allow attachment. The cells were then incubated with either 0.02% DMSO (Control) or 10 μM C6 ceramide (Treatment) for 48 h. Total RNA was extracted from the samples using TransZol reagent (TransGen Biotech, Beijing, China) following the manufacturer’s instructions. RNA libraries were constructed using rRNA-depleted RNAs, evaluated for quality and quantified using the Bioanalyzer 2100 system. Finally, the HiSeq sequencing platform (BGI Genomics, Shenzhen, China) was used for detection. The raw sequencing data, stored in the fastq format as raw data samples, underwent sequencing-related quality assessment. SeqPrep software and Sickle were utilized for quality control statistics, thereby obtaining high-quality clean data. HISAT was applied to align the clean data with the canine genome (Canis_familiaris), yielding mapped data (reads). StringTie was then used to assemble the mapped reads and compare them with existing genomic annotation information to discover new transcripts. Certain transcripts that were ≥200 bp in length, had at least two exons, and had an ORF (Open Reading Frame) length of ≤300 bp were selected as preliminary candidates for lncRNA. Then, software such as CPC (Coding Potential Calculator), CNCI (Codon Usage Bias), txCdsPredict, and Pfam were used for protein domain analysis to predict the coding capacity. Ultimately, we selected the intersection of these four software tools as the final lncRNA dataset. We used FPKM (Reads Per Kilobase of exon model per Million mapped reads) to estimate gene expression values and employed DESeq2 software for differential gene expression analysis between different groups. We set the selection criteria as |log_2_FC| ≥ 1 and *p* adjust < 0.05 to identify genes with significantly differential expression.

### 2.5. Quantitative Real-Time Polymerase Chain Reaction (qRT-PCR)

Total RNA was reverse-transcribed into complementary DNA (cDNA) using EasyScript^®^ All-in-One First-Strand cDNA (TransGen Biotech, Beijing, China). Quantitative real-time polymerase chain reaction (RT-qPCR) was performed using Taq pro-Universal SYBR qPCR Master Mix (Vazyme Biotech, Nanjing, China), and GAPDH was the reference gene. Each gene was subjected to three biological replicates. The primer sequences are shown in Table 1.

### 2.6. Nucleus-Cytoplasmic Separation

CHMp cells were plated at 3 × 10^5^ cells/well in 6-well plates and incubated overnight to allow attachment. A Cytoplasmic & Nuclear RNA Purification Kit (Norgen Biotek, Canada) was used to separate nuclear and cytoplasmic fractions from CHMp cells, according to the kit instructions. Each gene had three biological replicates.

### 2.7. Cell Transfection

Cells were plated in 6-well plates at 8 × 10^4^ cells/well and incubated overnight to allow attachment. The pcDNA3.1 Lnc_025370 overexpression plasmid was constructed using the pcDNA3.1 vector (Thermo Fisher Scientific, Waltham, MA, USA). Lipofectamine 3000 reagent (Thermo Fisher Scientific, Waltham, MA, USA) was used to transfect the plasmids into CHMp cells to establish Lnc_025370 overexpression in the CHMp cell (CHMp_Lnc_) and vector CHMp cell (CHMp_NC_) according to the manufacturer’s instructions. After transfection, the cells were collected and screened using a basic medium containing G418 for a minimum of five generations until stable cell lines were obtained. The stability of the CHMpLnc and CHMpNC cells was then verified by RT-qPCR.

### 2.8. Cell Growth Curves

Cell growth was quantified using a Cell Counting Kit-8 (Beyotime Biotechnology, Shanghai, China) to generate growth curves. CHMp_Lnc_ and CHMp_NC_ cells were plated in 96-well plates at 300 cells/well and incubated overnight to allow attachment. CCK-8 (10 μL) was added to each well 1, 2, 3, 4, 5, or 6 days later. Following 1 h of incubation, the absorbance was read at 450 nm with a microplate reader (Agilent Technologies, Santa Clara, CA, USA). Each group had five biological replicates.

### 2.9. Cell Migration Assay

Cell migration was determined by a wound-healing assay. CHMp_Lnc_ and CHMp_NC_ cells were plated in 6-well plates at 2 × 10^5^ cells/well and incubated overnight to allow attachment. A wound was created with a 200 μL pipette tip. After washing two times with PBS to remove cell fragments, cells were cultured with 2% FBS DMEM. Then, images of the wounds were photographed to evaluate cell migration. Image J 1.53c (National Institutes of Health, Bethesda, MA, USA) was used to analyze the migration rate. Each group had three biological replicates.

### 2.10. Invasion Assay

Matrigel^TM^ (BD Biosciences, CA, USA) was mixed with DMEM at a ratio of 1:30. Then, Matrigel (100 μL) was added to the upper chamber of 24-well transwell filters for 1h at 37 °C to the coated membrane. CHMp_Lnc_ and CHMp_NC_ cells were plated in 6-well plates at 2 × 10^5^ cells/well in the upper chamber with 100 μL FBS-free DMEM, and the lower chambers were filled with 500 μL complete DMEM with 10% FBS. Invading cells were fixed with 4% paraformaldehyde and stained with 0.1% (*w*/*v*) crystal violet, and photographed by a microscope (CKX41; Olympus), then counted with Image J 1.53c (National Institutes of Health, Bethesda, MA, USA). Each group had three biological replicates.

### 2.11. Western Blotting

Cells were plated in 6-well plates at 4 × 10^5^ cells/well and incubated overnight to allow attachment. With or without 6μM and 10 μM C6 ceramide treatment for 48 h, cells were lysed with RIPA buffer (Beyotime, Shanghai, China) supplemented with PMSF (Beyotime, Shanghai, China) on ice for 20 min. Proteins were quantified using the BCA protein assay kit (Beyotime, Shanghai, China). SDS-PAGE was used to separate total protein (20 μg) on a 10% gel, which was then transferred onto a PVDF membrane (IPVH00010, Milli-pore, Massachusetts, USA), and incubated with primary antibodies NRG1 (Abcam, UK, 1:1000) and GAPDH (Proteintech, Wuhan, China, 1:2500) overnight at 4 °C. HRP-conjugated anti-rabbit/mouse antibodies (Proteintech, Wuhan, China, 1:2000) were incubated for 1 h at room temperature. Finally, the membranes were visualized using a chemiluminescence imaging analysis system (Tanon 5200, Shanghai, China). Immunoblotting signals were quantified by densitometry using Image J 1.53c (National Institutes of Health, Bethesda, MA, USA).

### 2.12. Mouse Xenografts

Tumor xenografts were successfully established in 5-week-old BALB/c nude mice (SPF Biotechnology Co., Ltd., Beijing, China) through the subcutaneous implantation of CHMp_Lnc_ and CHMp_NC_ cells into the mammary fat pad (*n* = 5). A suspension of 5 × 10^6^ cells in 100 μL of phosphate-buffered saline (PBS) was used for each tumor inoculation. Monitoring of tumor progression, based on both tumor length and width, as well as the mice’s body weights, occurred on alternative days until the study’s conclusion on day 14. The tumor volume was determined using the formula: length × width^2^/2. Upon completion of the study, the mice were euthanized following anesthesia with isoflurane, followed by CO_2_ asphyxiation, to facilitate the collection of xenograft tumors. Tumor tissues were fixed with 10% neutral formalin and embedded in paraffin. Subsequently, the tissues were sectioned into serial slices, each 5 microns thick. Following this, the specimens were stained with hematoxylin and eosin (H&E) at room temperature for histopathological examination. For the evaluation of immuno-histochemical properties, the tissue sections were treated with an anti-Ki-67 antibody (Proteintech, Wuhan, China, 1:1400). All procedures involving animals were conducted with the approval of the Institutional Animal Care and Use Committee at Fujian Agriculture and Forestry University (Permit Number PZCASFAFU23006), ensuring ethical standards were strictly adhered to throughout the study.

### 2.13. Statistical Analysis

The data arepresented as the mean ± SD. A two-tailed unpaired Student’s *t*-test was used to evaluate the differences between the two groups. ANOVA with Tukey’s test for post hoc analysis was used to compare the differences among groups using GraphPad Prism software Version 8.0. * *p* < 0.05, ** *p* < 0.01, and *** *p* < 0.001 were considered significantly different.

## 3. Results

### 3.1. The Growth Inhibition and LncRNA Expression Profiles Under the Treatment of C6 Ceramide in Chmp Cells

**Figure 1:**
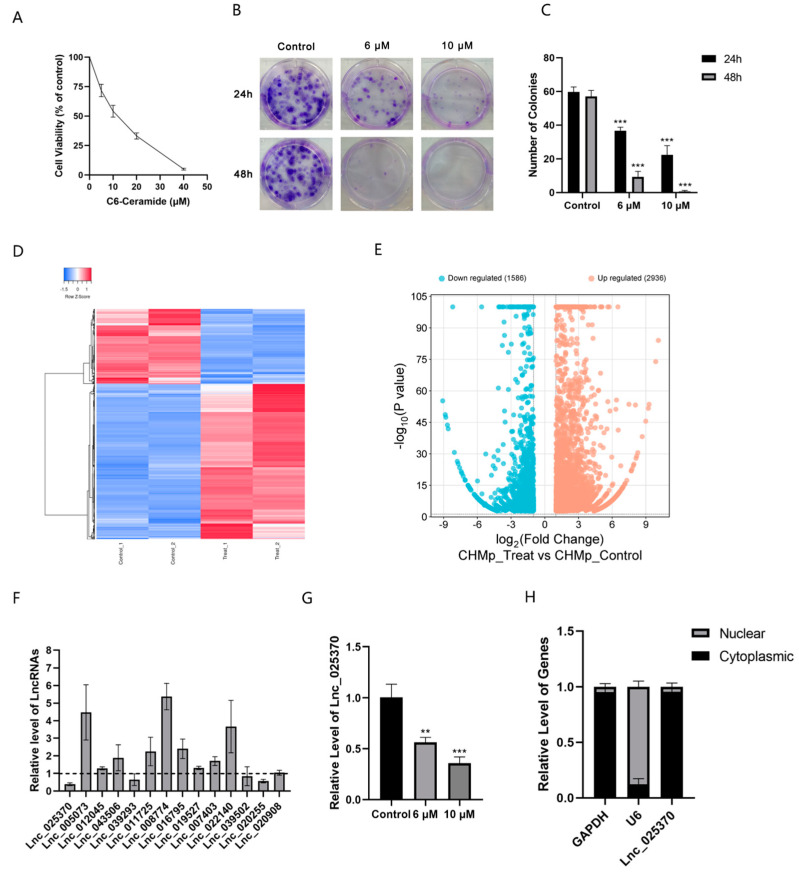
The growth inhibitory effect of C6 ceramide and its related lncRNAs in CHMp cells. (**A**) Cell viability was analyzed using CCK-8 in CHMp cells after exposure to C6 ceramide; (**B**,**C**) Colony formation of CHMp cells treated with C6 ceramide; (**D**) Heatmap plot of lncRNA. Red indicates high relative expression, blue indicates low relative expression; (**E**) Volcano plots of lncRNA expression; (**F**) Expression of lncRNAs by qRT-PCR; (**G**) Relative expression of Lnc_025370 after C6-ceramide treatment for 48 h; (**H**) Relative expression of Lnc_025370 in nuclear and cytoplasm of CHMp cells. The data are presented as the mean ± SD. ** *p* < 0.01, *** *p* < 0.001.

C6 ceramide was found to decrease the cell viability of CHMp cells in a dose-dependent manner (Figure 1A). The long-term effects of C6 ceramide on cell proliferation, as determined by colony formation assay, showed a significant reduction in clonogenic survival (Figure 1B,C). To investigate the potential roles of lncRNAs in C6 ceramide treatment, RNA sequencing was used to analyze the expression profiles of lncRNAs. The results revealed that under incubation with C6 ceramide, a total of 4522 significantly differentially expressed lncRNAs were identified, with 2936 lncRNAs upregulated and 1586 lncRNAs downregulated (Figure 1D,E). To confirm these findings, 14 lncRNAs exhibiting a significant fold change in expression were selected and subsequently validated through qRT-PCR. Among them, Lnc_025370 showed the lowest expression level, while Lnc_008774 showed the highest after treatment with C6 ceramide in CHMp cells (Figure 1F). Lnc_025370 was then chosen for further experiments to analyze its function and underlying mechanism. Furthermore, the expression of Lnc_025370 was examined after incubation with C6 ceramide, confirming it as a target of C6 ceramide. As shown in Figure 1G, C6 ceramide significantly decreased the expression of Lnc_025370 in a dose-dependent manner. Therefore, Lnc_025370 is one of the target agents of C6 ceramide. Subsequently, the subcellular localization of Lnc_025370 was determined, revealing its primary location in the cytoplasm (Figure 1H).

### 3.2. Overexpressing Lnc_025370 Promoted CHMp Cells Proliferation

**Figure 2:**
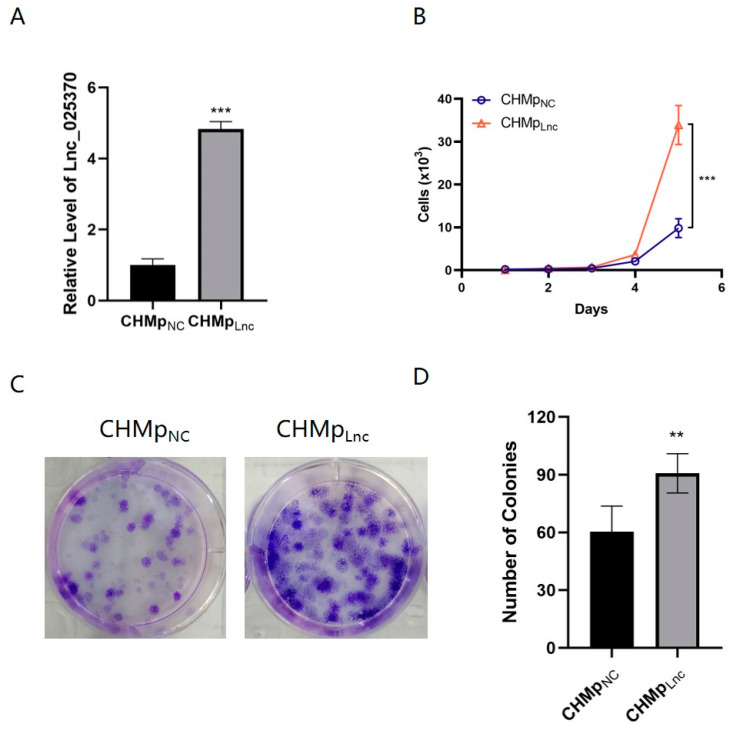
Overexpressing Lnc_025370 promoted CHMp cell proliferation. (**A**) The expression of Lnc_025370 and GAPDH was determined with qRT-PCR in cells transfected with the pcDNA3.1 vector (CHMp_NC_) and pcDNA3.1-Lnc_025370 (CHMp_Lnc_); (**B**) Growth curves of CHMp_NC_ and CHMp_Lnc_ cells; (**C**,**D**) Colony formation of CHMp_NC_ and CHMp_Lnc_ cells. The data are presented as the mean ± SD. ** *p* < 0.01, *** *p* < 0.001.

To further explore the function of Lnc_025370 in the progression of canine mammary carcinoma, we overexpressed Lnc_025370 in CHMp cells by an overexpression plasmid and named it CHMp_Lnc_, with the control group being named CHMp_NC_. Lnc_025370 expression was significantly greater in CHMp_Lnc_ cells compared to CHMp_NC_ cells (Figure 2A). There was a noticeable increase in the proliferation curve starting from the 5th day in CHMp_Lnc_ cells compared to CHMp_NC_ (Figure 2B). Similarly, we observed a higher colony formation in CHMp_Lnc_ cells compared to those in CHMp_NC_ cells (Figure 2C,D). Taken together, these data show that Lnc_025370 can exert a proliferative effect on CHMp cells.

### 3.3. Overexpressing Lnc_025370 Promoted CHMp Cells Metastasis

**Figure 3:**
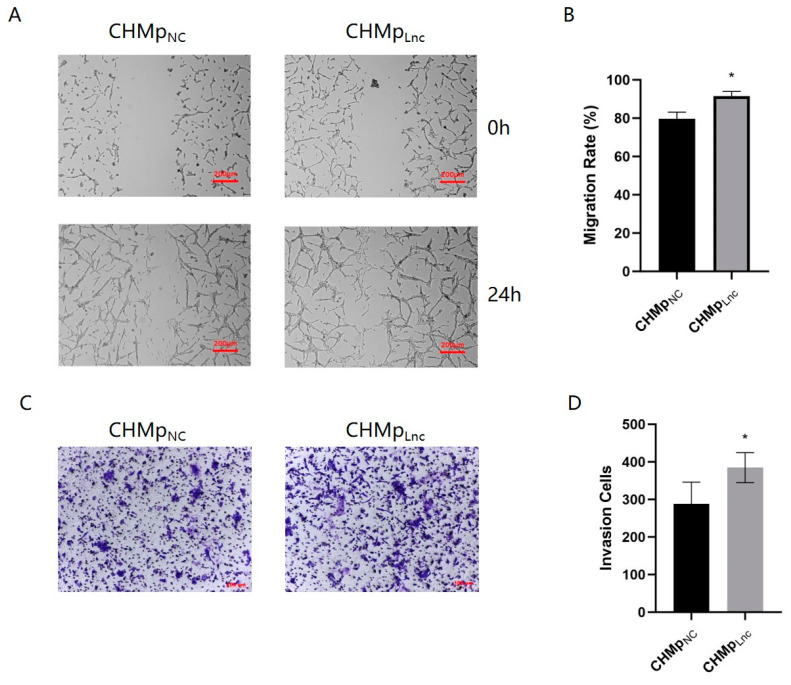
Overexpressing Lnc_025370 promoted CHMp cell metastasis migration and invasion. (**A**) The migratory ability of CHMp_NC_ and CHMp_Lnc_ cells. scale bar, 200 μm; (**B**) The migratory abilities of CHMp_NC_ and CHMp_Lnc_ were calculated by Image J. (**C**) Invading cells of CHMp_NC_ and CHMp_Lnc_ were stained with 0.1% (w/v) crystal violet. Scale bar, 100 μM; (**D**) The number of invading cells was calculated by Image J. The data are presented as the mean ± SD. * *p* < 0.05.

Migration and invasion are crucial factors in the metastasis of cancer cells. In CHMp_Lnc_ cells, the percentage of migration was 91.49% ± 2.49%, which was significantly higher than that in CHMp_NC_ cells, 79.74% ± 3.4% (Figure 3A,B). The transwell assay demonstrated a significant increase in the number of invading cells in the CHMp_Lnc_ cells (Figure 3C,D). These results demonstrate that Lnc_025370 effectively promotes metastasis in CHMp cells.

### 3.4. Overexpressing Lnc_025370 Attenuated Growth Inhibition of C6 Ceramide in CHMp Cells

**Figure 4:**
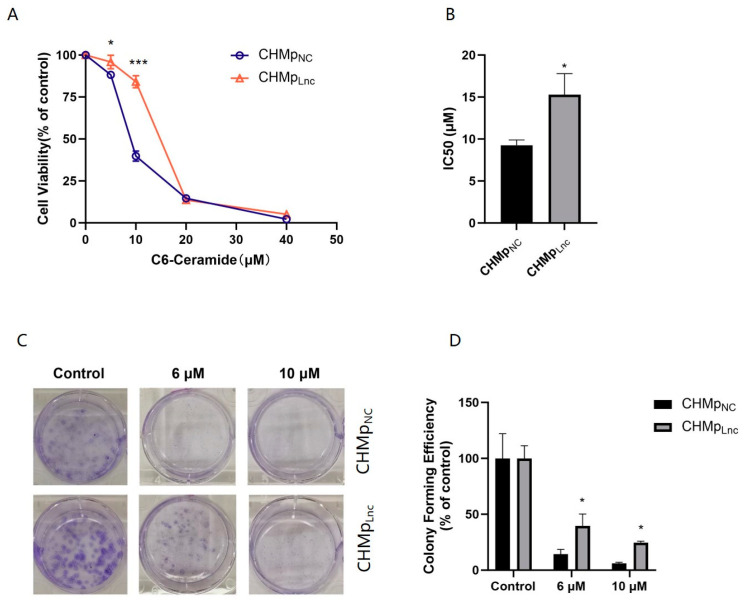
Overexpressing Lnc_025370 attenuated growth inhibition of C6 ceramide in CHMp cells (**A**) Cell viability was analyzed using CCK-8 in CHMp_NC_ and CHMp_Lnc_ cells after exposure to C6 ceramide for 48 h; (**B**) IC50 of C6-Ceramide on CHMp_NC_ and CHMp_Lnc_ cells; (**C**,**D**) Colony formation of CHMp_NC_ and CHMp_Lnc_ cells treated with C6 ceramide for 48 h. The data are presented as the mean ± SD. * *p* < 0.05, *** *p* < 0.001.

To identify whether Lnc_025370 is a target agent of C6 ceramide, we investigated the growth inhibition of C6 ceramide in CHMp_Lnc_ and CHMp_NC_ cells. As depicted in Figure 4A, the cell viability of CHMp_Lnc_ cells was significantly higher than that of CHMp_NC_ cells when treated with 5 μM and 10 μM C6 ceramide for 48 h. The IC50 values of C6 ceramide in CHMp_Lnc_ cells and CHMp_NC_ cells were 9.26 ± 0.62 μM and 15.29 ± 2.5 μM, respectively, displaying significant differences (Figure 4B). The results of colony formation were in agreement with the aforementioned findings, as colony formation in CHMp_Lnc_ cells increased compared to that in CHMp_NC_ cells after exposure to the same concentration of C6 ceramide (Figure 4C,D). These findings demonstrate that Lnc_025370 mitigates the growth inhibition caused by C6 ceramide in canine mammary carcinoma cells.

### 3.5. Overexpressing Lnc_025370 Attenuated Metastasis Inhibition of C6 Ceramide in CHMp Cells

**Figure 5:**
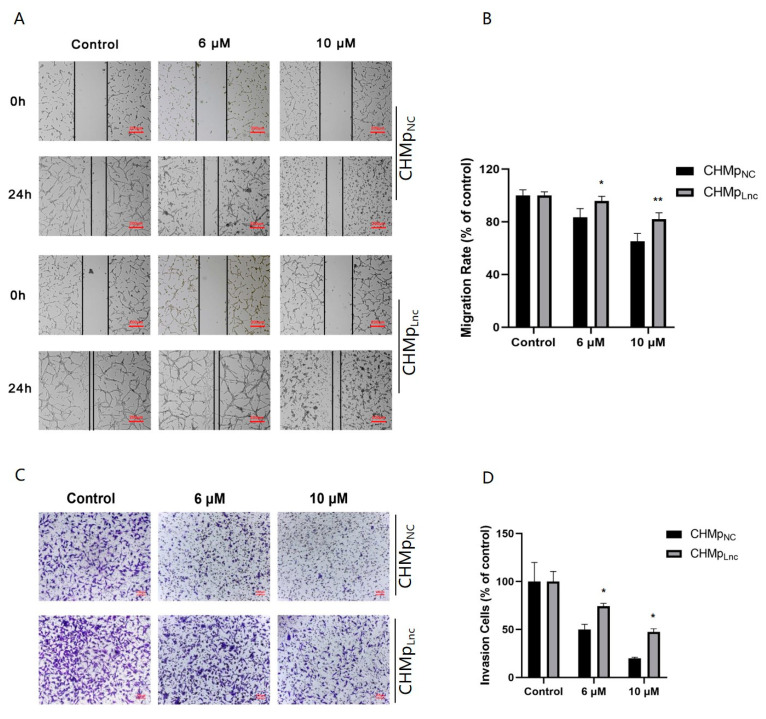
Overexpressing Lnc_025370 attenuated metastasis inhibition of C6 ceramide in CHMp cells. (**A**) The migratory ability of CHMp_NC_ and CHMp_Lnc_ cells after exposure to C6 ceramide for 24 h. scale bar, 200 μM; (**B**) The migratory abilities of CHMp_NC_ and CHMp_Lnc_ were calculated by Image J. (**C**) Invading cells of CHMp_NC_ and CHMp_Lnc_ were stained with 0.1% (w/v) crystal violet after exposure to C6 ceramide for 24 h. Scale bar, 100 μM; (**D**) The number of invading cells was calculated by Image J. The data are presented as the mean ± SD. * *p* < 0.05, ** *p* < 0.01.

To verify the effect of Lnc_025370 on C6 ceramide inhibiting the migration of CHMp, the wound healing assay and transwell assay were carried out. After treating the cells with C6 ceramide, we observed a significantly higher percentage of migration in CHMp_Lnc_ cells compared to CHMp_NC_ cells (Figure 5A,B). Moreover, the invasion abilities of CHMp_Lnc_ cells were considerably stronger than those of CHMp_NC_ cells when exposed to the same concentration of C6 ceramide (Figure 5C,D). These findings suggest that overexpressing Lnc_025370 weakens the inhibitory effect of C6 ceramide on metastasis in CHMp cells.

### 3.6. Lnc_025370 Promoted Xenograft Tumor Growth In Vivo

**Figure 6:**
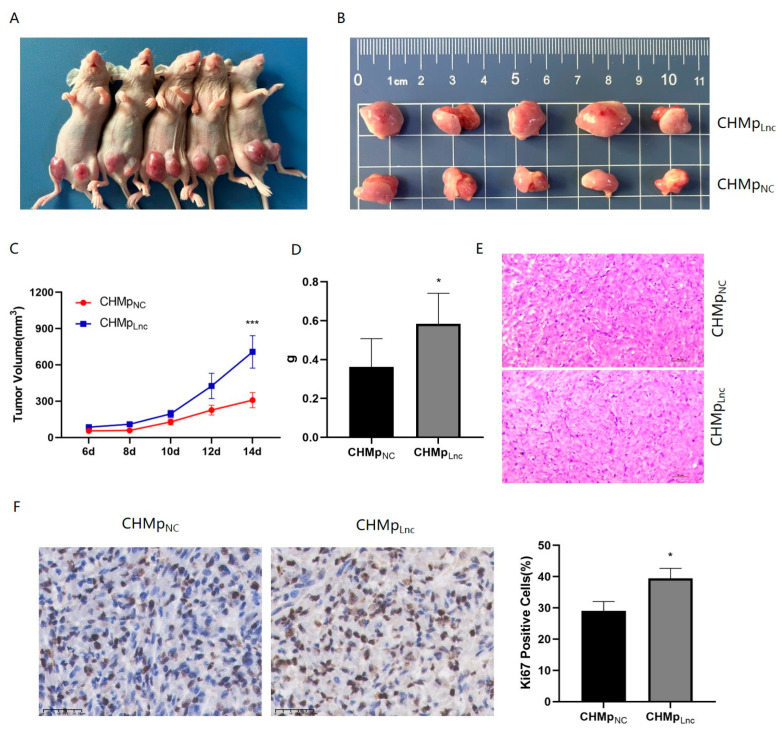
Lnc_025370 promoted xenograft tumor growth. (**A**) Mice with subcutaneously transplanted tumors; (**B**) Representative photographs of tumors after the experiment; (**C**) Fluctuations in the size of subcutaneously transplanted tumors across the groups; (**D**) Weight measurements of the subcutaneously transplanted tumors; (**E**) The pathological histology of the tumor masses (Scale bar = 50 μM); (**F**) Illustrative images of immunohistochemistry (IHC) depicting Ki67 expression (Scale bar = 50 μM), with a quantitative analysis of Ki67 staining corresponding to the images in both groups. The data are presented as the mean ± SD. * *p* < 0.05, *** *p* < 0.001.

To assess the impact of Lnc_025370 on the proliferation of CHMp cells in vivo, CHMp_NC_ and CHMp_Lnc_ cells were subcutaneously implanted into BALB/c nude mice to establish xenograft tumor models. After two weeks, the xenograft tumors were harvested (Figure 6A,B). On the 14th day, the tumor volume in the CHMp_Lnc_ group was substantially greater than that in the CHMp_NC_ group (Figure 6C), and the tumor weight was also significantly higher in the CHMp_Lnc_ group (Figure 6D). The pathological histology of the tumor masses derived from CHMp_NC_ and CHMp_Lnc_ cells is presented in Figure 6E, revealing a high number of poorly differentiated cells with pronounced atypia. Additionally, immunohistochemistry analysis using the proliferation marker Ki67 was conducted on the tumor tissues, and a significant difference was noted between the two groups (Figure 6F). These findings are consistent with our in vitro results and further validate the role of Lnc_025370 in promoting tumor growth in CHMp cells.

### 3.7. Lnc_025370 Promoted CHMp Cells Progress by Regulating NRG1

**Figure 7:**
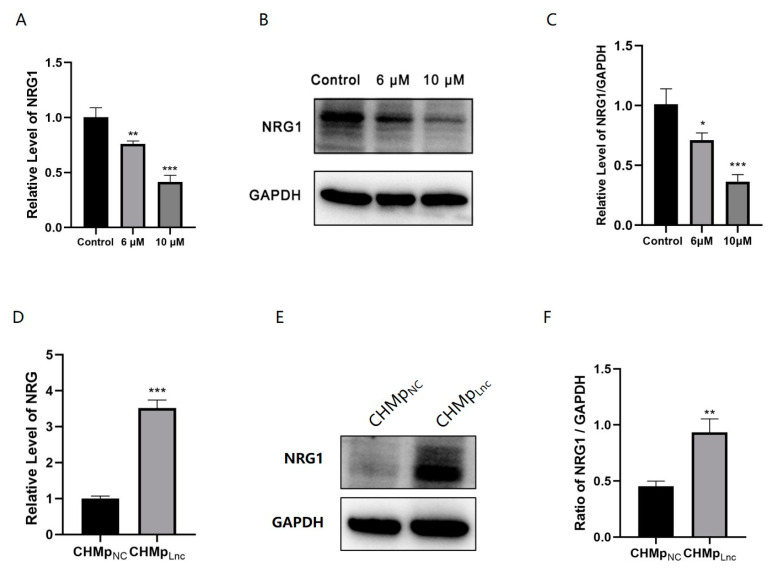
The promotion of Lnc_025370 in CHMp cells progression, with a possible association with NRG1 expression. (**A**) The expression of NRG1 and GAPDH was determined with qRT-PCR in CHMp cells after exposure to C6 ceramide for 48 h; (**B**,**C**) The expression of NRG1 was determined with western blotting in CHMp cells after exposure to C6 ceramide for 48 h; (**D**) The expression of NRG1 and GAPDH was determined with qRT-PCR in CHMp_NC_ and CHMp_Lnc_ cells; (**E**,**F**) The expression of NRG1 was determined with western blotting in CHMp_NC_ and CHMp_Lnc_ cells. The data are presented as the mean ± SD. * *p* < 0.05, ** *p* < 0.01, *** *p* < 0.001.

Cis-acting target gene prediction analysis revealed a potential regulatory relationship between NRG1 and Lnc_025370. After treatment with C6 ceramide, both the mRNA and protein levels of NRG1 were downregulated (Figure 7A–C), consistent with the trend observed in Lnc_025370. Additionally, the expression of NRG1 in CHMp_Lnc_ cells was significantly higher than in CHMp_NC_ cells (Figure 7D–F). These indicated that Lnc_025370 can upregulate the expression of NRG1, suggesting that Lnc_025370 may exert a cancer-promoting effect by activating NRG1.

## 4. Discussion

CMCs have posed a serious threat to canines’ lives and health for many years. Surgical treatment has limited effectiveness for metastatic tumors, necessitating the use of adjuvant chemotherapy. Unfortunately, veterinary clinics currently lack effective chemotherapy drugs, thereby highlighting the urgent need for discovering new treatment alternatives.

A large number of studies have reported the anti-tumor effects of ceramide. Whether it is endogenous ceramide induced by radiation treatment or chemotherapy medications, or exogenous ceramide artificially added, ceramides show greater toxicity towards tumor cells compared to normal cells and without visible toxicity in vivo [23,24]. C6 ceramide is a short-chain ceramide analog, which frequently plays an exogenous ceramide with anti-tumor properties, exerting an inhibitory effect on hepatocellular carcinoma [25], lymphoma [26], ovarian cancer [27], and thyroid cancer [28]. In human breast cancer, C6 ceramide has been demonstrated to inhibit cancer growth and metastasis through various mechanisms both in vivo and in vitro, either alone or in combination with other chemotherapeutic medications [29,30,31]. Spontaneous CMC serves as an ideal model for human breast tumors [3,12,13], C6 ceramide has a similar inhibition of growth and metastasis in CMC cells (Figure 1, Figure 4 and Figure 5). However, there is a lack of research on the lncRNAs associated with C6 ceramide. Therefore, we conducted a screening of the expression profiles of lncRNAs following treatment with C6 ceramide in CHMp cells. As a result, we identified a novel target agent of C6 ceramide, namely Lnc_025370. Subsequently, we demonstrated that Lnc_025370 is downregulated after treatment with C6 ceramide. Additionally, overexpression of Lnc_025370 was found to promote CHMp cell proliferation, colony formation efficiency, migration, and invasion, while weakening the inhibitory effect of C6 ceramide. These findings suggest that C6 ceramide targeting Lnc_025370 regulates CMC progression.

The diverse functions of lncRNAs are related to subcellular localization [32]. Studies have revealed that nuclear lncRNAs function by modulating transcriptional programs through chromatin interactions and remodeling [33,34]. Cytoplasmic lncRNAs are often described as translation regulators mediating mRNA and protein stability [35,36]. As lnc_025370 is mainly located in the cytoplasm (Figure 1H), it is worth investigating if its tumorigenic effect in CMC is related to any mRNA. Subsequently, analysis of cis-acting target genes predicted that NRG1 may be involved. NRGs are a group of signaling proteins primarily expressed in the nervous system, heart, and breast [37]. NRG1 belongs to the epidermal growth factor (EGF) family, which is crucial in linking hyperglycemic memory in breast cancer cells to malignant tumor progression [38]. Recent studies have shown that NRG1 is involved in the development of various tumors, promoting cell proliferation, invasion, and tumorigenesis. Furthermore, its overexpression is associated with invasive clinical features and a poor prognosis, making it a potential prognostic and therapeutic biomarker [39,40]. In our study, the expression of NRG1 was downregulated at both the mRNA and protein levels following treatment with C6 ceramide, and the overexpression of Lnc_025370 promoted the expression of NRG1 (Figure 6). These findings demonstrate that Lnc_025370 contributes to CHMp cells progression with a possible association with NRG1 expression, making it a potential therapeutic agent in CMC therapy.

Given the crucial role of lncRNAs in cancer regulation, their potential as biomarkers for the diagnosis and treatment of cancer in humans and dogs is enormous [22,41]. However, the functions of most lncRNAs have not been characterized, particularly in CMC. Further investigations may provide amazing insights for oncology or other pathological areas in veterinary medicine.

As a whole, our study identified 4522 differentially expressed lncRNAs following treatment with C6 ceramide in CHMp cells, which is beneficial in uncovering the novel mechanism of action of the anticancer agent C6 ceramide. Subsequently, we presented compelling evidence that LncRNA_025370 serves as a target for C6 ceramide, facilitating the growth and metastasis of CHMp cells through its association with NRG1. This suggests that targeting LncRNA_025370 could be a promising new approach for treating CMCs. There are, however, several limitations in this study. Firstly, the specific mechanism by which LncRNA_025370 regulates NRG1 remains unclear. Exploring its downstream molecules could help elucidate its functional mechanism. Secondly, the expression and functional validation of LncRNA_025370 in dog patients is lacking. It would be beneficial to conduct experiments using animal patients to confirm our findings. Lastly, it would be advantageous to investigate the expression of LncRNA_025370 in CMC patients and analyze its correlation with tumor histological grading and prognosis. Further studies are necessary to address these limitations and provide a more comprehensive understanding of the role of LncRNA_025370 in CMC.

## 5. Conclusions

This study revealed the lncRNA expression profile of C6 ceramide in CHMp cells and identified LncRNA_025370 as a target molecule of C6 ceramide. Furthermore, we have confirmed that LncRNA_025370 promotes the growth and metastasis of CHMp cells, and this is associated with an increase in the expression of NRG1. These findings indicate that LncRNA_025370 could potentially serve as a novel approach for the treatment of CMCs.

## Figures and Tables

**Table 1:** The primers for RT-qPCR.

Genes	Sequence (5’→3’)
Lnc_025370	Forward: GTAGTGGCAATCCAAGAAGGAG Reverse: AAAGTCATACCAGGCAAGAAGG
Lnc_005073	Forward: CTGCACCTCCTCCATACTTACC Reverse: CCTGAGCAATACCTGTCTCTCC
Lnc_012045	Forward: TGCCAACTAAAACACAGGTCAG Reverse: ACATGGCAGTGGGATGAAAG
Lnc_043506	Forward: ACTCCCTTGGCTACCAGATAC Reverse: CGTCAAGAAGGACTTTAAGCAG
Lnc_039293	Forward: TATATGCTGGCCAAAGCTCAC Reverse: TAGCACAGTGTTTTCCTGGTTC
Lnc_011725	Forward: ATGCGCTCTCTCTCTCTCTCTC Reverse: GATTCCAGGCCACTCTAATCTC
Lnc_008774	Forward: GGGAGCTGTACTGTGGGTTC Reverse: TGACCCTGTATGGCTCTGTG
Lnc_016795	Forward: GCAAACATCATAGAAGGGGATG Reverse: GGAGAACATTTGGCAGAGAGAG
Lnc_019527	Forward: TGACTGATGACTACGAGGAAGG Reverse: TTTAAGTCGCGTGCTCTGTG
Lnc_007403	Forward: TGAAATCAGTGTCCCAAGAGG Reverse: AACCTGGAACCCACAGAGTTAG
Lnc_022140	Forward: AGGCCTCGGTTCCTTAAGAC Reverse: ACACGCCGAGAAGTTTCATT
Lnc_039502	Forward: TAGTGAATTCGGGCATACAGG Reverse: GCCAACGATGTATTCAACAGG
Lnc_020255	Forward: CGTTGCATCCTCTGGTTGTA Reverse: GCAGATCGGAGTTGAGAAGG
Lnc_020908	Forward: TCAGGAGCAAGTGAAGAGTAGG Reverse: AATGGACAGACGACAGGTACAG
NRG1	Forward: ATGGTTCAAGAACGGGAATG Reverse: CGATGGTGATATTGGCAGAG
GAPDH	Forward: ATGTTTGTGATGGGCGTGAA Reverse: GCTAGAGGAGCCAAGCAGTT
U6	Forward: AAGATGGCGGACAAAGAG Reverse: CTCTGTCAGCTTGTACTGGTC

## Data Availability

The original contributions presented in the study are included in the article; further inquiries can be directed to the corresponding authors. The datasets for this study can be found in NCBI’s SRA database (BioProject ID: PRJNA1065941).
